# The nature and content of rumination for head and neck cancer survivors

**DOI:** 10.3389/fpsyg.2022.995187

**Published:** 2022-10-26

**Authors:** Fiona Menger, Jennifer Deane, Joanne M. Patterson, Peter Fisher, James O’Hara, Linda Sharp

**Affiliations:** ^1^Speech and Language Sciences, School of Education, Communication and Language Sciences, Newcastle University, Newcastle-upon-Tyne, United Kingdom; ^2^Population Health Sciences Institute, Newcastle University, Newcastle-upon-Tyne, United Kingdom; ^3^School of Health Sciences, University of Liverpool, Liverpool, United Kingdom; ^4^Newcastle upon Tyne Hospitals NHS Foundation Trust, Newcastle-upon-Tyne, United Kingdom

**Keywords:** rumination, head and neck (H&N) cancer, cancer surviorship, post-traumatic growth, post-traumatic stress disorder, cancer

## Abstract

**Introduction:**

Head and neck cancer (HNC) diagnosis and treatment can be a significant life trauma. Some HNC survivors experience post-traumatic growth (PTG), which has been linked with better health-related quality-of-life. Empirical research on PTG, and theoretical models, point to the importance of being able to purposely make sense of the traumatic experience. Intrusive rumination, by contrast, is linked to poorer outcomes. This study explored HNC survivors’ experiences of rumination.

**Methods:**

Twenty HNC survivors between 9 months and 5 years post-diagnosis were recruited (11 male, 9 female, age range 46–83). They had a range of HNC sub-types and cancer treatments. Participants underwent a semi-structured interview about their cancer diagnosis and treatment. Reflexive thematic analysis identified themes and sub-themes around rumination.

**Results:**

Four themes with linked subthemes on the content and process of rumination were identified. Theme 1 was *rumination and worry related to diagnosis*. Here, survivors discussed how the HNC diagnosis and plans for treatment had dominated their initial thoughts. Theme 2 was *processing the trauma of HNC*. This theme reflected rumination on the traumatic experience of diagnosis and treatment and how the participant was reacting to it. Theme 3 was *considering the impact*. This theme encompassed retrospective thinking (e.g., on treatment decisions made) and comparisons between the participant now versus the early days after diagnosis. Theme 4 was *continued rumination*. This theme included ongoing intrusive and distressing rumination about the trauma and impact of cancer. Those who expressed ongoing rumination revisited fears (e.g., concerns about their future) or returned to negative experiences (e.g., distressing exchanges with healthcare professionals or what they perceived as poor care).

**Conclusion:**

This study uniquely describes the nature and content of rumination following HNC. Early intrusive rumination is common and may reflect perceptions of cancer as an existential threat. Over time, rumination can become more reflective and move towards deliberate meaning-making. Some HNC survivors may benefit from interventions to reduce barriers to this transition. The content of distressing and difficult to control rumination (commonly focused on ongoing fears or inability to resolve difficult experiences) helps to identify those who may benefit from more directed psychological support.

## Introduction

Head and neck cancer (HNC) is a significant life trauma (Bingo et al., 2020). Combined, cancers of the lips, oral cavity, larynx, pharynx, and salivary glands are the 8^th^ most common cancer worldwide (Sung et al., 2021) and incidence rates are rising (Cancer Research UK, 2022). People from deprived socioeconomic backgrounds are disproportionately affected (Taib et al., 2018) and diagnosis is often made at a late stage.

It can be argued that the emotional and psychological trauma of HNC is unique. There are often negative consequences for quality of life (Qualizza et al., 2019). Because of the location of tumours, treatment for the cancer is often arduous and multimodal and can lead to ongoing alteration of vital functions of everyday life such as speech, voice, and swallowing (Patterson et al., 2013; Rogers et al., 2016). Survivors are likely to have many medical appointments and will have multiple interactions with healthcare professionals. Living with the condition often presents ongoing trauma and distress (Björklund et al., 2010). Survivors may be unable to work for a period, or permanently (Pearce et al., 2015). Moreover, they experience poor psychosocial adjustment (Buchmann et al., 2013; Clarke et al., 2014), high rates of distress, and significant levels of depression and anxiety (De Leeuw et al., 2000). This patient group has high rates of pre-existing mental illness and substance abuse, and many have restricted access to social support (Rieke et al., 2016). Incidence of suicide is also significantly higher relative to survivors of other cancers (Jansen et al., 2018). Consequently, there is a recognized need for targeted psychosocial support (Scott et al., 2013; Smith et al., 2017). However, high quality evidence informing the development of interventions towards improving psychosocial outcomes is poor (Semple et al., 2015). Interventions such as CBT, which have been effective in other cancers, do not successfully reduce distress for the HNC population and there is limited to no evidence base to support interventions aimed at improving quality of life (Calver et al., 2018).

Accumulating evidence suggests many cancer survivors may experience post-traumatic growth (PTG) (Shand et al., 2015; Casellas-Grau et al., 2017). Those who do have been shown to have better health-related quality of life; (Ruini et al., 2013; Liu et al., 2020) Health-related quality of life has been identified as a prognostic indicator of survival (Ediebah et al., 2018). Focused quantitative studies suggest that PTG is also experienced following HNC, (Holtmaat et al., 2017; Harding, 2018; Sharp et al., 2018) and these experiences are described within qualitative reports (Ruf et al., 2009; Thambyrajah et al., 2010; Menger et al., 2022). However, levels of PTG have been reported to be lower in HNC than in other cancers (Sharp et al., 2018). Some psychological interventions have shown promise in relation to supporting the development of PTG in cancer survivors (Li et al., 2020). However, they have not yet been tested in those with HNC who may, arguably, be considered a more challenging patient population with specific needs. Moreover, while the available qualitative and quantitative studies on PTG in HNC provide valuable insight into factors that may influence PTG, and describe how it is experienced in this group, the extent to which they can inform the development of interventions is limited, as they do not shed light on how and why some cancer survivors–but not others–progress from experience of trauma and distress towards the recognized PTG outcomes, i.e., greater appreciation of life, changed relationships, personal strength, new possibilities, and spiritual growth (Menger et al., 2021).

The revised model on development of PTG provided by Tedeschi et al. (2018) acknowledges influences such as the person-pre trauma, the centrality of the event, disruption of core beliefs, sociocultural influences, and the management of emotional distress and coping. Rumination following trauma is central to the model. Therefore, its role in relation to the development of PTG following cancer has been of interest to applied cancer researchers seeking to identify the best means to provide psychological support. Existing research recognizes the possibility that the earlier stages of rumination and perseverative thinking are not problematic and allow natural adaptation to occur (Wells, 2000; Wells and Sembi, 2004). Rumination may only become problematic when it becomes difficult to control.

Quantitative research (across cancer types) suggests that the ability to purposely make sense of traumatic experiences and to find meaning amidst challenges are important following the trauma of diagnosis and treatment (Wang et al., 2016; Ogińska-bulik and Kobylarczyk, 2019). Conversely, intrusive rumination/a focus on negative thoughts and feelings has been related to post-traumatic stress and poorer outcomes (Caspari et al., 2017; Zhang et al., 2018; Ogińska-bulik and Kobylarczyk, 2019). Qualitative studies on cancer survivorship present findings that investigate lived experiences across diagnosis and treatment pathways and beyond. Themes related to the existence of rumination and experiences of PTG frequently occur across this literature but are not overtly addressed: for example, distressing thoughts (Stenhammar et al., 2017), recognition of self-awareness as a coping mechanism (Walshe et al., 2017), and moves towards redemption and psychological change (Scrignaro et al., 2018). Qualitative studies focused broadly on HNC survivorship also touch upon rumination, e.g., the importance of meaning-making and introspection (Threader and Mccormack, 2016; Grattan et al., 2018), and individual needs to reduce perseverative thinking styles (Calver et al., 2018). However, studies specifically focusing on rumination in HNC survivors are lacking.

Understanding why rumination, worry, doubting, and over-analysis continues for HNC survivors is of interest if effective psychological interventions are to be developed. It is, therefore, important to have insight into the nature and content of rumination experienced. A key action in the development of interventions is to obtain a thorough understanding of service user contexts and user experiences, particularly in contexts where any future interventions may be implemented. Therefore, better understanding of how rumination and reflective behaviours manifest would help towards building theory on pathways to PTG and contribute towards a “framework of actions” for intervention development (O’Cathain et al., 2019). This could help identify means to promote PTG following HNC and also identify and support those who develop cancer-related post-traumatic stress symptoms (PTSS).

Given the unique characteristics of the HNC patient group and the many related sequalae that may trigger ruminative thinking, the above literature (and broader literature on rumination in other traumas) is insufficient to illuminate the nature and content of rumination for a HNC population. This study, therefore, aimed to explore ruminative thinking following HNC. From this, the hope is to better understand how rumination may influence the development of PTG following HNC, and how PTSS are maintained.

## Materials and methods

This qualitative study focused on cancer survivors who have lived through HNC diagnosis and subsequent treatment and who hold unique understanding of those experiences.

### Participants

Participants were recruited *via* HNC outpatient lists at a specialist ENT centre. All had received a diagnosis of HNC and were at least 9 months and up to 5 years after completion of treatment. This time-period was chosen to allow time for a period of reflection post-diagnosis and treatment that was sufficient to allow any PTG to develop (Tedeschi et al., 2018) but not beyond a period where participants may have struggled to recall details of their experiences.

### Data collection

Semi-structured interviews took place either on the telephone or within a private office. The interviewer (FM) used a topic guide developed in collaboration with a cancer research patient and public involvement group and a person who had received treatment for head and neck cancer. The topic guide was influenced by Tedeschi et al.’s (2018) revised model of PTG. It was designed to elicit data that might illuminate pathways towards PTG; the interviewer encouraged participants to return to their cancer diagnosis and treatment by discussing how they coped with their experiences, how they felt about living with head and neck cancer, and whether anything positive had come out of their situation. Interviews were audio recorded and transcribed.

A reflexive thematic analysis approach was chosen, guided by the approach of Braun and Clarke (2019a). This allowed for inductive analysis that would capture themes from patient recollections of diagnosis and treatment. Data collection ceased at twenty participants, when the interviewer felt transcripts contained sufficient diversity of experience and relevance in relation to the research questions to have reached ‘information power’ (Malterud et al., 2016; Braun and Clarke, 2019b).

### Data analysis

Initial data familiarization took place by reading and re-reading transcripts and by checking back to the original recordings. Two researchers (FM & JD) individually coded the first five transcripts using specialized software (NVivo V.12). This involved highlighting areas of the text which were felt to be related to rumination or reflection on the HNC cancer experience. The remaining 15 transcripts were split between the two researchers to code independently; they then met regularly to discuss the emerging coding framework. Once completed, each set of transcripts was then reviewed by the other researcher to discuss and resolve any remaining discrepancies. This approach supported quality of coding by allowing for ‘situated, reflexive interpretation’ (Braun and Clarke, 2019b) as researchers were immersed in the data during a period of intense collaboration. Once coding was completed, the primary author brought together codes which reflected overarching themes on the type of ruminative thinking described and sub-themes reflecting “shared patterns of meaning” on the nature of those thought processes. By taking this approach, subjectivity is acknowledged as a core assumption, but also a strength of reflexive thematic analysis, which recognizes researchers as a resource towards construction of themes from qualitative data (Braun and Clarke, 2022).

## Results

**Figure 1:**
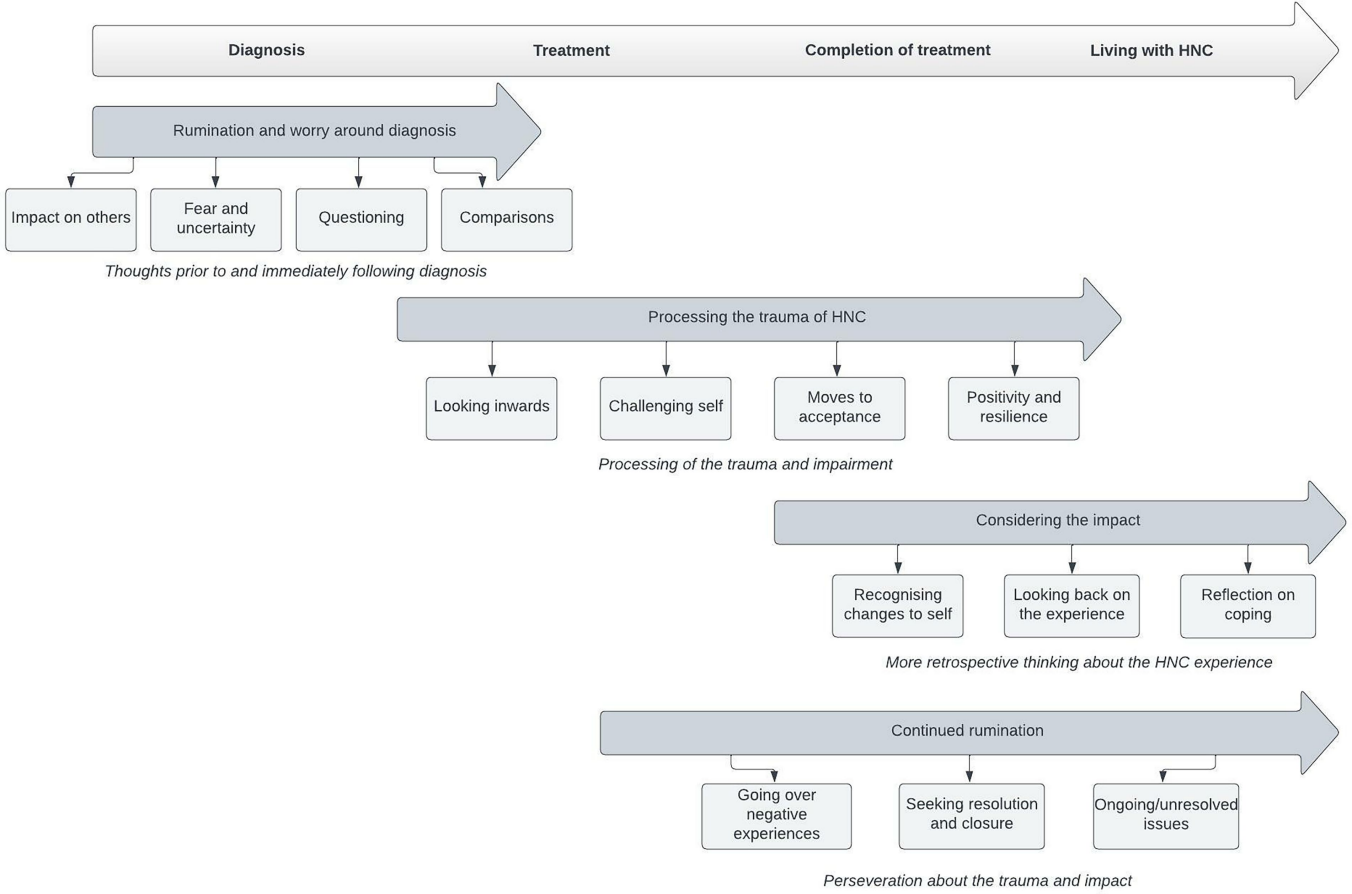
Temporal visualization of themes related to rumination in HNC survivors.

Twenty participants took part in the study, demographics are outlined in Table 1. Four main themes were identified following analysis, with related subthemes (Figure 1). Theme 1 was *rumination and worry around diagnosis* (subthemes: impact on others, fear and uncertainty, questioning, comparisons). Theme 2 was *processing the trauma of HNC* (subthemes: looking inwards, challenging self, moves to acceptance, positivity, and resilience). Theme 3 was *considering the impact* (subthemes: recognizing changes to self, looking back on the experience, reflection on coping). Theme 4 was *continued rumination* (subthemes: going over negative experiences, seeking resolution and closure, ongoing unresolved issues). Each of the themes are described below, using pseudonyms for participant quotes.

**Table 1:** Participant characteristics.

Characteristic	*n*				
**Sex**		**Cancer directed treatment**		**Cancer site**	
Female	11	Surgery	15	Neck	3
Male	9	Radiotherapy	15	Base of tongue	6
		Chemotherapy	8	Tonsil	5
**Age at interview**		**HPV Status**		Larynx	2
40–49	1	Positive	14	Nasopharynx	1
50–59	7			Salivary gland	1
		**T stage at diagnosis**			
60–69	8	T0/T1	10	Hypopharynx	1
70+	4	T2	8	Parotid	1
		T3/T4	2		

### Rumination and worry around diagnosis

In this theme, survivors discussed how the HNC diagnosis and plans for treatment had dominated their initial thoughts.

*Impact on others*: Some participants discussed experiencing intrusive thoughts about the impact of their situation on those close to them; in the early stages of either realizing there was a medical problem that needed to be investigated or in the light of the HNC diagnosis, they commonly focused their thoughts on the impact if they were to die from cancer.

*You think about, you know, the stage of life you are at. You're very much aware of the impact it* [the cancer diagnosis] *has on the people around you and those people that you love… so it makes a pretty heady cocktail to have to react and come to terms with.* Martin

“*I think you sort of run ahead of it, and it was about the death and the dying. And I had three, sort of, quite young teenagers then, and I just wanted to live until* [youngest child] *was eighteen.”* Victoria

*Fear and uncertainty*: Fearful thoughts about an unclear future dominated, as participants recounted how they entered *“some pretty dark places”* (Martin) and would dwell on what might happen, often exploring every possible outcome, including possible consequences of treatment and their own death. Jackie said, “*I was very emotional and very scared,”* while Doreen gave more insight into the precise nature of her early thoughts, “*Oh, I would expect it to be, I’d be losing hair, I would be awful, losing my weight, I’m not able to… I had a terrible dark imagination*.”

*Questioning*: Early ruminative thinking often questioned why HNC had happened and what might be the possible cause. “*It was really quite a shock, because* [I had] *no family history and I wondered if it was connected to the immune system or something”* (Doreen). Some dwelled upon lifestyle causes. “*I smoked and drunk quite a lot when I was younger. I blamed that. They did tell me it wasn’t from smoking or anything, but it does not help.. I still blamed myself for the cancer.”* (Bianca).

*Comparisons*: During the early stages of treatment, participants commonly described comparing their own situation with others. This sometimes focused on people who did not have cancer, or on those who could do the things in life they could not. Most commonly, participants described how they made comparisons between themselves and other cancer patients. Thoughts were around how others were worse off or worries about how a fellow patient was faring. For example, Bianca discussed a person she met during her treatment:

*I think it was just I so many times wanted to go back up on to the ward when I was going for my reviews just to see if he’s still here. But then I didn’t know how I would feel if… I think he was going to stop his treatment because he knew there was nothing they could do for him. It was just really pain, I think, relief that they were going to be giving him after that.*

Although most comparison was downward, one participant (Victoria) made upward comparisons, reporting she often felt annoyed or exasperated at others with less traumatic cancer experiences or who told her about other problems in their lives, feeling she had to ‘*bite her tongue’* not to make the point that what she had experienced was worse.

### Processing the trauma of HNC

This theme concerned the trauma of cancer diagnosis and treatment and reaction to participants’ experiences. After rumination on the uncertainty a diagnosis of HNC brings, coupled with fear and uncertainty about many aspects of life, many described moving towards thinking about how the diagnosis and treatment could have had a positive impact.

*Looking inwards*: When forced to spend a lot of time thinking about cancer and what it would mean for them, some spoke of how they spent time focusing on their own character and how they responded to the situation, e.g., Martin said “*… it’s in those quieter moments a lot of it at night asleep or awake you learn to come to terms, as in come to terms as a result of any therapeutic intervention or reliance on others because ultimately it’s you. You have to deal with it.”* Several reported that this type of thinking happened at night or when alone.

Some experienced a sense of disconnection, struggling to see themselves as someone who was unwell or needed help. Victoria, who had worked in hospitals, resisting seeing herself as a patient, saying,” *…when you go into the radiotherapy area, and it was always, “I’m not supposed to be here. I do not know why I’m here”* Others acknowledged the threat of cancer to themselves but chose not to focus thoughts on possible negative outcomes, e.g., Billy observed “*I do not know whether that’s just in my psyche, but I can honestly say all through the whole thing I never felt afraid. I never thought I was going to die. I never dwelt on anything like that.”*

*Challenging self*: The experience of cancer treatment also brought opportunities for self-questioning. Participants spoke about questioning how they wanted to spend the remainder of their lives, their faith, and their relationships. Clive came to recognize that he had previously behaved unsupportively towards his partner, *“I was in the radiotherapy, wasn’t really strapped down that long but things like that go through your mind, you are thinking about it on and off. It is just something you realize, you know….”*

*Moves to acceptance:* As treatment progressed, some participants spoke of deliberate rumination towards acceptance of their situation, including that they might die from cancer. For example, as Jackie said, “*You know, thinking about, if I do die, what…? You know, nice things, and how nice it was to have children, and things like that, yeah.”* There was recognition of being fortunate, despite ongoing difficulties:

*I don't feel heartbroken about not being able to sing* [anymore] *but I sometimes think, I just want to… you're listening to your favourite tune, and you want to sing along. I whistle along with great enthusiasm. That's another minor thing…But I mean I'm alive, I'm fit and well, so I don't mope about any of these things. I had brilliant treatment and I survived it.* Tania

*Positivity and resilience:* Many participants thought about the need for positivity throughout the treatment process, spending time thinking about the need to move away from negative emotions. Kenneth spoke about this when he felt himself challenged, “*I think I just took a positive attitude throughout. I was in this situation, and I just had to make the best of it and get through it.”* Participants spoke of recognizing their own strength through positivity, e.g., Mel’s determination. “*Well, you have got to be positive. It’s no good moaning, you have got to have a positive attitude. I thought I was going to do these things. I’m still going to see my grandkids again, simple as that.”* This deliberate positivity was also seen in inward self-motivation, e.g., Joyce talked about her resolution after a difficult night dealing with side effects, “*Sometimes if I’ve got a little bit of a cold, things are really nasty. I get up in the morning and I just think, “Thank goodness I can get up and go to work and forget about all this.”*

### Considering the impact

Reflection on specific aspects of the cancer experience was common, with this theme and subtheme focused on how participants described their ongoing questioning and self-analysis.

*Recognising changes in self:* Participants recognized that, following their experience, some things about themselves had changed and indeed their thought processes had changed:

*I think in the quiet moments when I was on my own, I did think about it and I thought well, I hope this all comes back and* [I] *recover my face, and that's my only brush with cancer. I hope that I don't – nothing else comes up but that is in the back of your mind. I don't feel a thing now, I have to say. I feel in a good place now and I feel now that I'm coming up to the second year of that, I don't feel so affected by it.* Janice

Changes to thought processes also came about because of shifts in belief systems, e.g., a previously held belief that cancer would lead to death and increase in awareness shifting that belief.

*I think, when I was a child, you know, one or two people that we knew – like, you know, family members or close friends – got cancer, and it was just a death sentence. So, everyone I knew who had got cancer… and that could well be because I didn’t hear about people getting it and recovering. To me, it was always a death sentence.* Jackie

*Looking back on the experience:* Much reflective thinking was focused on looking back at the experience of treatment for HNC, recalling the difficult journey, the traumatic aspects, treatment decisions that were made, and how expectations met the reality of treatment. For example, Billy said, “*Just out of the blue he* [oncologist/consultant] *just told me he was going to try me on this different drug, which was Cetuximab. I think that’s what saved my life.”* Reflecting on a more negative experience during radiotherapy and offering suggestions for improvement, Katherine said, “*Maybe to be told a little bit more because when I did go for my appointment, for my mask* [which holds the person’s head in place while they have radiotherapy]*, they said I was the second or the third person not to be told what I was actually going for that day. I found that a bit horrendous.* Katherine.

*Reflection on coping*: For some participants, there was also personal reflection on how they had reacted to the cancer: for example, their role in decision-making and good vs. bad decisions they had made during the experience:

*I think in hindsight I made the right choice. He said if you ever had to go back in again, that the scar tissue on the original cut that he had to make, that skin would be toughened, it would be a little bit more difficult to separate all the nerves to be able to get back in there. I just thought I'm not going to go for that. His advice would be he wouldn't go for that, but it was entirely my choice.* Janice

Participants also reflected on their behaviour towards others at the time. Mick told how the treatment effects had made him self-conscious of his looks and as a result more distant with people, saying’ “*I have a collar which I wear. I am extremely self-conscious of.. one person has seen me wearing the mask and that was by mistake. I’m irritated that happened. So, in some ways it’s forced me back in on myself a little bit.”* Bianca recalled how she had physically distanced herself from members of a multidisciplinary team by asking for more personal space following her realisation that so many people in one room must have meant a very serious problem. Tony looked back on his decision not to tell his daughters (who had previously lost their mother to cancer) that he was also suffering from the disease. This decision had led to him masking difficulties with eating and drinking and eventual hospitalization due to malnutrition:

*Yes, just trying to protect them, in hindsight now, looking back, I suppose it’d be different if it hadn’t happened to their mam and stuff, it probably wouldn’t be as bad, but it was because I can still feed myself, I didn’t want a feeding tube, all that type of thing, in hindsight I should have done because it would have kept me stronger because that last week I went downhill…*

For some, reflection involved comparisons between their situation during treatment and now. “*When you have been sitting there with tubes up your nose and you could not drink and you could not eat, this is a million per cent better because I can drink now, and I can eat now. So, it’s a good result, as far as I’m concerned”* (Kenneth).

### Continued rumination

This final theme included intrusive and distressing rumination about the trauma and impact of cancer. Although this phase was not reported by all participants, it was common and, when reported, provided clear insight into the ongoing nature of negative rumination containing distressing content.

*Going over negative experiences*: Some intrusive thoughts involved returning to negative experiences. For example, Mick was still dwelling on interactions with a charity organization he felt failed to provide him with support, while Billy was able to recount in detail an interaction with a doctor who delivered bad news in an insensitive way. Bianca recounted a distressing experience when someone had drawn attention to her radiation scars in public:

*… we all know, you can walk along the street, and you see somebody with either a disfigurement or something wrong with them and yes, you tend to look… you try and stop yourself because you’re trying to not make somebody feel so bad. But to shout something across an open space, it wasn’t like I was standing right beside him or anything like that, and just go, “What have you done to your neck?” or something like that. It obviously still bothers me.*

*Seeking resolution and closure:* There were participants who discussed ongoing thoughts about situations they felt needed to be resolved. For example, Jackie was seeking affirmation for decisions she had made about her own care, saying she felt she would have been more comfortable about her decision to pursue complementary treatment routes had she been offered an appropriate person to talk to who had been down the same route.

In Kenneth’s case, the ongoing need for resolution related to a legal action over early failures of the healthcare system, which he felt had missed his diagnosis; he remained angry and emotional, feeling a need for justice and retribution. Although he initially said he had “*come to terms with it,”* he later stated that he had not. He expressed ongoing anger and loss, saying he was keen that others should hear his story and that “*I need them involved to be exposed one way or another.”*

*Ongoing/unresolved issues:* Some participants discussed ongoing physical problems. Although they had recovered well, they still noticed differences to how they were before, e.g., Katherine recalled how someone had asked if she had a sore throat, “*No I have not. It’s just obviously the way things have turned out, there’s a big change in my voice*.” These types of changes acted as an ever-present reminder of the HNC experience.

*I can eat anything I like, and I know I’m lucky in that. I can speak. Sometimes get the tickle at the back of my throat and think I’m choking, and I always need more water than anybody else, have a lot of phlegm. But my neck is never going to change. I’ve got pain in my right shoulder from a damaged accessory nerve. I know it’s trivial, but it’s a constant reminder that will never go away.* Victoria

Some participants needed resolution related to possible treatments and a belief that there was still more that might be done for them:

*I sometimes wonder whether… there may be some avenues that remain unexplored and it's about signposting where might I want to go next. I'm sometimes sure that maybe I haven't seen the end of the level of support that is available for me from the team who do what they do.* Martin

Finally, some participants’ responses indicated they had uncertainty about their futures, i.e., a fear of spread or of recurrence of their cancer. “*You cannot help but think about it. I do not care who they say that can, it’s always in the back of your mind. Will it come back? Even now you are thinking it will come back again*” (Mel).

## Discussion

This research set out to explore the nature of rumination after HNC. The resulting analysis provides unique insight into how HNC survivors ruminate upon the experience of cancer and into how rumination changes over time. A focus on the existential threat of cancer is common in the early stages after diagnosis. This frequently gives way to a process of meaning making. Ongoing rumination, when individuals return to aspects of their diagnosis and treatment that were unhelpful or disruptive, follows; survivors can also continue to ruminate on treatment sequelae or unresolved aspects of their care.

The nature of rumination following HNC does appear to be temporal as people first focus thoughts on what is to come and later look back on those experiences or dwell upon what feels unresolved. However, intrusive ‘what if’ thoughts and worries can also occur at any point, most notably fear of spread/recurrence or the consequences of ongoing sequalae. This in keeping with the revised model of PTG (Tedeschi et al., 2018) and the theory that, following trauma, individuals need time to ruminate for growth to occur. Our participants were deliberately recruited at least 6 months after completion of their treatment. That they had time to look back on their experiences is clearly reflected in our findings.

Early intrusive rumination related to the threats imposed by HNC was a very common response to diagnosis and plans for treatment. This is unsurprising, given societal narratives about cancer and related treatments as something to fear. HNC, when suspected or identified, presents a sudden deviation from what is described by the Common-Sense model as ‘the normative self.’ (Leventhal et al., 2016). Representations of threats to health that are activated as illness representations include predictions on the duration or consequences of a condition as well as pre-existing beliefs about treatment or cure (Hale et al., 2007). Our findings reflect these beliefs and public perceptions about cancer, which are present amongst the participants’ early intrusive ruminations. Pre-existing beliefs about cancer (and HNC in particular) as an existential threat dominated people’s thoughts. As survivors navigated this new and unknown experience, those beliefs were challenged as more reflection on what had happened in comparison with what they anticipated. This phase of rumination may be what either directly or indirectly leads a HNC survivor towards aspects of PTG.

Some may find this journey easier to navigate than others, while others may experience a longer period of experiencing threat and suffering before reaching a point of psychological change (Seiler and Jenewein, 2019). An increase in symptoms is related to more intrusive rumination, as shown by previous work on colon cancer (Thomsen et al., 2013); this is reflected in our findings as participants with HNC-related sequalae discussed the ongoing consequences of these difficulties on their lives and their thought patterns. Some, however, had come to a point of resolution where they no longer dwelt on the cancer and had been able to move forward. This ability to reappraise and find meaning and purpose in the HNC experience is likely to be key in the development of PTG. Of note, the current study did not attempt to determine which participants had developed PTG or were on a route to do so, nor to make comparisons within the group. Our previous work with this dataset (Menger et al., 2022) reported that most participants had moderate to high scores on the PTG Inventory short form (Cann et al., 2010). The participants’ ability to ruminate on how HNC diagnosis and treatment were handled is therefore reflective of the fact that most had attempted to find meaning in the experience. They did so by inwardly evaluating their response to specific aspects of their cancer journey, recognizing unhelpful thoughts and behaviors and adapting positively; they also acknowledged how their beliefs about HNC had been challenged.

The ability to reach a point of resolution following trauma is clearly beneficial; part of this involves letting go of the circumstances that are beyond an individual’s control, something often influenced by internal and external factors (Curran et al., 2017). Variations in individual coping styles in HNC survivors (Sherman et al., 2000; List et al., 2002; Derks et al., 2005) are likely to play a role. Other important factors may be gender differences in rumination (Johnson and Whisman, 2013), levels of distress (Holtmaat et al., 2017), and external factors such as availability of social support (Kim and Son, 2021; Menger et al., 2022). How these factors interact and when HNC survivors make meaningful transitions towards growth would be better illuminated by longitudinal studies, which are currently lacking but would be of particular value in this field.

One outcome of this work should be to inform those aiming to develop interventions towards encouraging PTG following HNC. The current findings suggest that researchers should now focus on where barriers and opportunities exist along the care pathway to support HNC patients to tell their stories and reflect upon their journey. Cancer survivors do not necessarily want access to formal psychological support beyond the early stages of diagnosis, preferring the presence of family and friends and valuing the ability to speak openly about their cancer (Richardson et al., 2015). For those who do not have good access to practical and emotional support, interventions such as peer befriending could be effective in reducing distress and offering opportunity for self-disclosure (Hu et al., 2019; Hatton et al., 2022). Other researchers are exploring the power of narratives to encourage better psychological outcomes, (e.g., Wise et al., 2018).

The therapeutic benefit of participating in qualitative research such as this should not be disregarded. Murray (2003) discusses how connection and rapport with a skilled and non-judgmental interviewer offers a therapeutic opportunity. Indeed, several of our participants commented that they found the experience useful. By telling the story of their treatment, participants were able to look back reflectively; others have shown that this can lead to self-revelations (Drury et al., 2007). Participation in research may also offer opportunity to find meaning in the cancer experience and ‘give back’ (Grattan et al., 2018).

It is also vital to identify what aspects of HNC care and the linked negative thoughts trigger rumination. Leventhal et al. (2016) discuss the importance of preparedness for treatment within healthcare settings. Our participants experienced ongoing negative thoughts related to circumstances where they felt misinformed or uncertain. Treatments for HNC are often arduous and multi-modal; for instance, some patients may have combinations of cancer-directed surgery, chemotherapy, and radiotherapy. Survivors can be left with sequalae that are distressing and lifelong, and these sequalae are sometimes unexpected by the patient. Of course, not everything can be certain (or predicted) in cancer care, and not every patient wishes to be informed about the possible negative consequences of treatment. Longitudinal studies may shed more light on whether advance knowledge of possible distressing treatment effects following HNC is supportive of more positive reflections at a later stage.

Our study also reinforces a need to identify those who may have experiences on which they dwell or feel unable to resolve (Nik Jaafar et al., 2022), or those for whom there have been clinical incidents or conflicts with the care team. One participant’s experience of early misdiagnosis and subsequent need for resolution had led to a prolonged period of intrusive rumination and anger over their experiences; in contrast, those who felt they had had good experiences of care, such as shared decision making, reflected positively upon the experience. Better social support following cancer–which could come from friends, family, and/or health professionals–is related to PTG (Sharp et al., 2018). Where this is strong, interactions with others may act to facilitate more deliberate rumination and self-disclosure. Conversely, conflict, negative interactions, additional burden, or unresolved issues within the recovery environment may trigger ongoing and intrusive thinking. To proactively avoid such conflicts, healthcare professionals may benefit from training in interactions which could facilitate deliberate rumination; this is work on improving services that could be designed *via* processes of user involvement (Attree et al., 2010).

Metacognitive therapy (MCT, Wells, 2000) could be valuable for those most at risk from poorer psychological outcomes. The MCT model proposes that metacognitive beliefs, i.e., beliefs about cognition such as “I cannot stop ruminating,” “ruminating will help me to problem solve” prevent people from moving on from traumatic memories, images, and thoughts. MCT has been applied to cancer survivors in small scale studies (Fisher et al., 2017, 2019) and resulted in the reduction of emotional distress including clinically significant post-traumatic stress symptoms. Cancer survivors who engaged with the therapy (which was most participants) found it acceptable and useful to support understanding of unhelpful rumination and prevent becoming ‘locked’ into unhelpful thinking (Wells and Sembi, 2004). This type of intervention is supportive of the development of adaptive coping and resilience, which is related to better life satisfaction after cancer (Adamkovič et al., 2022).

This study was somewhat limited in that recruitment came from a single specialist center and there were only 20 participants. However, the group did represent diverse experiences. A further limitation is the cross-sectional design; a participant’s current situation may influence how they recall past events and experiences. Future studies could explore rumination concurrent to points in HNC pathways and beyond completion of treatment.

To conclude, this study provides a unique insight into the experiences of rumination for HNC survivors and how that rumination relates to cancer diagnosis and treatment. The findings support understanding of how some HNC survivors look back upon their cancer journey in a way that can be facilitative of PTG and how others experience ongoing and intrusive rumination. They shed light on the content both of rumination that is common but likely to be transitory following HNC diagnosis and treatment and later continued, negative rumination which may place survivors at risk of post-traumatic stress and poor psycho-social outcomes. In the early stages of survivorship, patients may benefit from supportive interactions and environments to enable them to successfully process the trauma of cancer diagnosis and treatment. Those experiencing later ongoing rumination may benefit from metacognitive interventions towards the reduction of emotional distress.

## Data availability statement

The data that support the findings of this study are available from the corresponding author upon reasonable request.

## Ethics statement

The studies involving human participants were reviewed and approved by NHS North of Scotland Research Ethics Service. The patients/participants provided their written informed consent to participate in this study.

## Author contributions

LS, JO’H, JP, and FM contributed to conception and design of the study. LS, JP, and JO'H secured finding. JO'H facilitated recruitment. FM carried out the interviews. FM and JD conducted the analysis. PF consulted on psychology aspects of the work. FM wrote the first draft of the manuscript with support from LS. All authors contributed to the article and approved the submitted version.

## Funding

This research was supported by funding from the Newcastle upon Tyne Hospitals Charity (Award no. BH172327).

## Conflict of interest

The authors declare that the research was conducted in the absence of any commercial or financial relationships that could be construed as a potential conflict of interest.

## Publisher’s note

All claims expressed in this article are solely those of the authors and do not necessarily represent those of their affiliated organizations, or those of the publisher, the editors and the reviewers. Any product that may be evaluated in this article, or claim that may be made by its manufacturer, is not guaranteed or endorsed by the publisher.

## References

[ref1] Adamkovič M. Fedáková D. Kentoš M. Bozogáňová M. Havrillová D. Baník G.. (2022). Relationships between satisfaction with life, posttraumatic growth, coping strategies, and resilience in cancer survivors: a network analysis approach. Psychooncology 1–9. doi: 10.1002/pon.5948, PMID: 35524705 PMC9790334

[ref2] Attree P. Morris S. Payne S. Vaughan S. Hinder S. (2010). Exploring the influence of service user involvement on health and social care services for cancer. Health Expect. 14, 48–58. doi: 10.1111/j.1369-7625.2010.00620.x, PMID: 20673242 PMC5060558

[ref3] Bingo S. A. M. Maree J. E. Jansen van Rensburg J. J. M. (2020). Living with cancer of the head and neck: a qualitative inquiry into the experiences of South African patients. Eur. J. Cancer Care (Engl). 29:e13205. doi: 10.1111/ecc.13205, PMID: 31829489

[ref4] Björklund M. Sarvimäki A. Berg A. (2010). Living with head and neck cancer: a profile of captivity. J. Nurs. Healthc. Chronic Illn. 2, 22–31. doi: 10.1111/j.1752-9824.2010.01042.x

[ref5] Braun V. Clarke V. (2019a). Reflecting on reflexive thematic analysis. Qual. Res. Sport. Exerc. Heal. 11, 589–597. doi: 10.1080/2159676X.2019.1628806

[ref6] Braun V. Clarke V. (2019b). To saturate or not to saturate? Questioning data saturation as a useful concept for thematic analysis and sample-size rationales. Qual. Res. Sport. Exerc. Heal. 13, 201–216. doi: 10.1080/2159676X.2019.1704846

[ref7] Braun V. Clarke V. (2022). Thematic Analysis a Practial Guide. London, Sage.

[ref8] Buchmann L. Conlee J. Hunt J. Agarwal J. White S. (2013). Psychosocial distress is prevalent in head and neck cancer patients. Laryngoscope 123, 1424–1429. doi: 10.1002/lary.23886, PMID: 23553220

[ref9] Calver L. Tickle A. Moghaddam N. Biswas S. (2018). The effect of psychological interventions on quality of life in patients with head and neck cancer: a systematic review and meta-analysis. Eur. J. Cancer Care (Engl). 27, e12789–e12718. doi: 10.1111/ecc.12789, PMID: 29094780

[ref10] Cancer Research UK (2022) Head and neck cancer incidence trends over time. Available at: https://www.cancerresearchuk.org/health-professional/cancer-statistics/statistics-by-cancer-type/head-and-neck-cancers/incidence#heading-Two (Accessed June 10, 2022).

[ref11] Cann A. Calhoun L. G. Tedeschi R. G. Taku K. Vishnevsky T. Triplett K. N.. (2010). A short form of the posttraumatic growth inventory. Anxiety Stress Coping 23, 127–137. doi: 10.1080/10615800903094273, PMID: 19582640

[ref12] Casellas-Grau A. Ochoa C. Ruini C. (2017). Psychological and clinical correlates of posttraumatic growth in cancer: a systematic and critical review. Psychooncology 26, 2007–2018. doi: 10.1002/pon.4426, PMID: 28317221

[ref13] Caspari J. M. Raque-Bogdan T. L. McRae C. Simoneau T. L. Ash-Lee S. Hultgren K. (2017). Posttraumatic growth after cancer: the role of perceived threat and cognitive processing. J. Psychosoc. Oncol. 35, 561–577. doi: 10.1080/07347332.2017.1320347, PMID: 28414581

[ref14] Clarke S. A. Newell R. Thompson A. Harcourt D. Lindenmeyer A. (2014). Appearance concerns and psychosocial adjustment following head and neck cancer: a cross-sectional study and nine-month follow-up. Psychol. Heal. Med. 19, 505–518. doi: 10.1080/13548506.2013.855319, PMID: 24215497

[ref15] Curran L. Sharpe L. Butow P. (2017). Anxiety in the context of cancer: a systematic review and development of an integrated model. Clin. Psychol. Rev. 56, 40–54. doi: 10.1016/j.cpr.2017.06.003, PMID: 28686905

[ref16] De Leeuw J. R. J. De Graeff A. Ros W. J. G. Blijham G. H. Hordijk G. J. Winnubst J. A. M. (2000). Prediction of depressive symptomatology after treatment of head and neck cancer: the influence of pre-treatment physical and depressive symptoms, coping, and social support. Head Neck 22, 799–807. doi: 10.1002/1097-0347(200012)22:8<799::AID-HED9>3.0.CO;2-E, PMID: 11084641

[ref17] Derks W. De Leeuw J. R. J. Hordijk G. J. Winnubst J. A. M. (2005). Differences in coping style and locus of control between older and younger patients with head and neck cancer. Clin. Otolaryngol. 30, 186–192. doi: 10.1111/j.1365-2273.2004.00938.x, PMID: 15839873

[ref18] Drury V. Francis K. Chapman Y. (2007). Taming the rescuer: the therapeutic nature of qualitative research interviews. Int. J. Nurs. Pract. 13, 383–384. doi: 10.1111/j.1440-172X.2007.00654.x, PMID: 18021168

[ref19] Ediebah D. E. Quinten C. Coens C. Ringash J. Dancey J. Zikos E.. (2018). Quality of life as a prognostic indicator of survival: a pooled analysis of individual patient data from Canadian cancer trials group clinical trials. Cancer 124, 3409–3416. doi: 10.1002/cncr.31556, PMID: 29905936

[ref20] Fisher P. L. Byrne A. Fairburn L. Ullmer H. Abbey G. Salmon P. (2019). Brief metacognitive therapy for emotional distress in adult cancer survivors. Front. Psychol. 10, 1–8. doi: 10.3389/fpsyg.2019.00162, PMID: 30766505 PMC6365419

[ref21] Fisher P. L. Byrne A. Salmon P. (2017). Metacognitive therapy for emotional distress in adult cancer survivors: a case series. Cognit. Ther. Res. 41, 891–901. doi: 10.1007/s10608-017-9862-9, PMID: 29104332 PMC5656708

[ref22] Grattan K. Kubrak C. Caine V. O’Connell D. A. Olson K. (2018). Experiences of head and neck cancer patients in middle adulthood: consequences and coping. Glob. Qual. Nurs. Res. 5:233339361876033. doi: 10.1177/2333393618760337, PMID: 29568793 PMC5858616

[ref23] Hale E. D. Treharne G. J. Kitas G. D. (2007). The common-sense model of self-regulation of health and illness: how can we use it to understand and respond to our patients’ needs? Rheumatology 46, 904–906. doi: 10.1093/rheumatology/kem060, PMID: 17449488

[ref24] Harding S. (2018). Positive psychological change in head and neck cancer populations. J. Cancer Treat. Diagnosis 2, 1–7. doi: 10.29245/2578-2967/2018/2.1126 29030020

[ref25] Hatton R. A. Crane J. Rogers S. N. Patterson J. (2022). Head and neck cancer peer-to-peer support and quality of life: systematic scoping review. Br. J. Nurs. 31, S30–S36. doi: 10.12968/bjon.2022.31.5.S30, PMID: 35271361

[ref26] Holtmaat K. van der Spek N. Cuijpers P. Leemans C. R. Verdonck-de Leeuw I. M. (2017). Posttraumatic growth among head and neck cancer survivors with psychological distress. Psychooncology 26, 96–101. doi: 10.1002/pon.4106, PMID: 26918531

[ref27] Hu J. Wang X. Guo S. Chen F. Wu Y. Yu J.. (2019). Peer support interventions for breast cancer patients: a systematic review. Breast Cancer Res. Treat. 174, 325–341. doi: 10.1007/s10549-018-5033-2, PMID: 30600413

[ref28] Jansen F. Verdonck-de Leeuw I. M. Cuijpers P. Leemans C. R. Waterboer T. Pawlita M.. (2018). Depressive symptoms in relation to overall survival in people with head and neck cancer: a longitudinal cohort study. Psychooncology 27, 2245–2256. doi: 10.1002/pon.4816, PMID: 29927013 PMC6231089

[ref29] Johnson D. P. Whisman M. A. (2013). Gender differences in rumination: a meta-analysis. Pers. Individ. Dif. 55, 367–374. doi: 10.1016/j.paid.2013.03.019, PMID: 24089583 PMC3786159

[ref30] Kim H. Son H. (2021). Moderating effect of posttraumatic growth on the relationship between social support and quality of life in colorectal cancer patients with ostomies. Cancer Nurs. 44, 251–259. doi: 10.1097/ncc.0000000000000887, PMID: 33886236 PMC8081094

[ref31] Leventhal H. Phillips L. A. Burns E. (2016). The common-sense model of self-regulation (CSM): a dynamic framework for understanding illness self-management. J. Behav. Med. 39, 935–946. doi: 10.1007/s10865-016-9782-2, PMID: 27515801

[ref32] Li J. Peng X. Su Y. He Y. Zhang S. Hu X. (2020). Effectiveness of psychosocial interventions for posttraumatic growth in patients with cancer: a meta-analysis of randomized controlled trials. Eur. J. Oncol. Nurs. 48:101798. doi: 10.1016/j.ejon.2020.101798, PMID: 32688246

[ref33] List M. A. Rutherford J. L. Stracks J. Haraf D. Kies M. S. Vokes E. E. (2002). An exploration of the pretreatment coping strategies of patients with carcinoma of the head and neck. Cancer 95, 98–104. doi: 10.1002/cncr.10653, PMID: 12115322

[ref34] Liu Z. Doege D. Thong M. S. Y. Arndt V. (2020). The relationship between posttraumatic growth and health-related quality of life in adult cancer survivors: a systematic review. J. Affect. Disord. 276, 159–168. doi: 10.1016/j.jad.2020.07.044, PMID: 32697695

[ref35] Malterud K. Siersma V. D. Guassora A. D. (2016). Sample size in qualitative interview studies: guided by information power. Qual. Health Res. 26, 1753–1760. doi: 10.1177/1049732315617444 26613970

[ref36] Menger F. Deane J. Patterson J. M. Fisher P. O’Hara J. Sharp L. (2022). “Post-traumatic growth after head and neck cancer: a qualitative comparison of survivorship experiences,” in preparation

[ref37] Menger F. Mohammed Halim N. A. Rimmer B. Sharp L. (2021). Post-traumatic growth after cancer: a scoping review of qualitative research. Support Care Cancer 29, 7013–7027. doi: 10.1007/s00520-021-06253-2, PMID: 34018030 PMC8464569

[ref38] Murray B. L. (2003). Qualitative research interviews: therapeutic benefits for the participants. J. Psychiatr. Ment. Health Nurs. 10, 233–236. doi: 10.1046/j.1365-2850.2003.00553.x, PMID: 12662341

[ref39] Nik Jaafar N. R. Hamdan N. A. Abd Hamid N. Rajandram R. K. Mahadevan R. Zakaria H.. (2022). Posttraumatic growth and its association with unmet supportive care needs and fear of cancer progression among head and neck cancer patients. PLoS One 17:e0265502. doi: 10.1371/journal.pone.0265502, PMID: 35290419 PMC8923508

[ref40] O’Cathain A. Croot L. Duncan E. A. S. Rousseau N. Sworn K. Turner K.. (2019). Guidance on how to develop complex interventions to improve health and health care. BMJ Open 9:e0265502. doi: 10.1136/bmjopen-2019-029954, PMID: 31420394 PMC6701588

[ref41] Ogińska-bulik N. Kobylarczyk M. (2019). The role of rumination in posttraumatic growth in people struggling with cancer. J. Psychosoc. Oncol. 37, 652–664. doi: 10.1080/07347332.2019.1600628, PMID: 31084411

[ref42] Patterson J. M. McColl E. Carding P. N. Hildreth A. J. Kelly C. Wilson J. A. (2013). Swallowing in the first year after chemoradiotherapy for head and neck cancer: clinician and patient-reported outcomes. Head Neck 36, 352–358. doi: 10.1002/hed 23780908

[ref43] Pearce A. Timmons A. O’Sullivan E. Gallagher P. Gooberman-Hill R. Thomas A. A.. (2015). Long-term workforce participation patterns following head and neck cancer. J. Cancer Surviv. 9, 30–39. doi: 10.1007/s11764-014-0382-2, PMID: 25060809

[ref44] Qualizza M. Bressan V. Rizzuto A. Stevanin S. Bulfone G. Cadorin L.. (2019). Listening to the voice of patients with head and neck cancer: a systematic review and meta-synthesis. Eur. J. Cancer Care (Engl). 28:e12939. doi: 10.1111/ecc.12939, PMID: 30284763

[ref45] Richardson A. E. Morton R. Broadbent E. (2015). Psychological support needs of patients with head and neck cancer and their caregivers: a qualitative study. Psychol. Health 30, 1288–1305. doi: 10.1080/08870446.2015.1045512, PMID: 25925706

[ref46] Rieke K. Boilesen E. Lydiatt W. Schmid K. K. Houfek J. Watanabe-Galloway S. (2016). Population-based retrospective study to investigate preexisting and new depression diagnosis among head and neck cancer patients. Cancer Epidemiol. 43, 42–48. doi: 10.1016/j.canep.2016.06.008, PMID: 27391545

[ref47] Rogers S. N. Heseltine N. Flexen J. Winstanley H. R. Cole-Hawkins H. Kanatas A. (2016). Structured review of papers reporting specific functions in patients with cancer of the head and neck: 2006-2013. Br. J. Oral Maxillofac. Surg. 54, e45–e51. doi: 10.1016/j.bjoms.2016.02.012, PMID: 26923873

[ref48] Ruf M. Buchi S. Moergeli H. Zwahlen R. A. Jenewein J. (2009). Positive personal changes in the aftermath of head and neck cancer diagnosis: a qualitative study in patients and their spouses. Head Neck 31, 513–520. doi: 10.1002/hed.21000 19132723

[ref49] Ruini C. Vescovelli F. Albieri E. (2013). Post-traumatic growth in breast cancer survivors: new insights into its relationships with well-being and distress. J. Clin. Psychol. Med. Settings 20, 383–391. doi: 10.1007/s10880-012-9340-1, PMID: 23229823

[ref50] Scott D. A. Mills M. Black A. Cantwell M. Campbell A. Cardwell C. R.. (2013). Multidimensional rehabilitation programmes for adult cancer survivors. Cochrane Database Syst. Rev. 2016:CD007730. doi: 10.1002/14651858.CD007730.pub2, PMID: 23543556 PMC6457813

[ref51] Scrignaro M. Marini E. Magrin M. E. Borreani C. (2018). Emotive and cognitive processes in cancer patients: linguistic profiles of post-traumatic growth. Eur. J. Cancer Care 27:e12620. doi: 10.1111/ecc.12620, PMID: 28058742

[ref52] Seiler A. Jenewein J. (2019). Resilience in cancer patients. Front. Psych. 10:208. doi: 10.3389/fpsyt.2019.00208, PMID: 31024362 PMC6460045

[ref53] Semple C. Parahoo K. Norman A. McCaughan E. Humphris G. Mills M. (2015). Psychosocial interventions for patients with head and neck cancer. Int. J. Evid. Based Healthc. 13, 37–38. doi: 10.1097/XEB.0000000000000015 25734867

[ref54] Shand L. K. Cowlishaw S. Brooker J. E. Burney S. Ricciardelli L. A. (2015). Correlates of post-traumatic stress symptoms and growth in cancer patients: a systematic review and meta-analysis. Psychooncology 24, 624–634. doi: 10.1002/pon.3719, PMID: 25393527

[ref55] Sharp L. Redfearn D. Timmons A. Balfe M. Patterson J. (2018). Posttraumatic growth in head and neck cancer survivors: is it possible and what are the correlates? Psychooncology 27, 1517–1523. doi: 10.1002/pon.4682, PMID: 29473248

[ref56] Sherman A. C. Simonton S. Adams D. C. Vural E. Hanna E. (2000). Coping with head and neck cancer during different phases of treatment. Head and Neck 22, 787–793. doi: 10.1002/1097-0347(200012)22:8<787::AID-HED7>3.0.CO;2-R, PMID: 11084639

[ref57] Smith J. D. Shuman A. G. Riba M. B. (2017). Psychosocial issues in patients with head and neck cancer: an updated review with a focus on clinical interventions. Curr. Psychiatry Rep. 19, 56–11. doi: 10.1007/s11920-017-0811-9, PMID: 28726060

[ref58] Stenhammar C. Isaksson J. Granström B. Laurell G. Ehrsson Y. T. (2017). Changes in intimate relationships following treatment for head and neck cancer—a qualitative study. J. Psychosoc. Oncol. 35, 614–630. doi: 10.1080/07347332.2017.1339224, PMID: 28605311

[ref59] Sung H. Ferlay J. Siegel R. L. Laversanne M. Soerjomataram I. Jemal A.. (2021). Global cancer statistics 2020: GLOBOCAN estimates of incidence and mortality worldwide for 36 cancers in 185 countries. CA Cancer J. Clin. 71, 209–249. doi: 10.3322/caac.21660 33538338

[ref60] Taib B. G. Oakley J. Dailey Y. Hodge I. Wright P. du Plessis R.. (2018). Socioeconomic deprivation and the burden of head and neck cancer—regional variations of incidence and mortality in Merseyside and Cheshire, north west. England. Clin. Otolaryngol. 43, 846–853. doi: 10.1111/coa.13067, PMID: 29341454

[ref61] Tedeschi R. G. Shakespeare-Finch J. Taku K. Calhoun L. G. (2018). Posttraumatic Growth. Theory, Research, and Applications. Abingdon, Oxon: Routledges

[ref62] Thambyrajah C. Herold J. Altman K. Llewellyn C. (2010). “Cancer doesn’t mean curtains”: benefit finding in patients with head and neck cancer in remission. J. Psychosoc. Oncol. 28, 666–682. doi: 10.1080/07347332.2010.516812, PMID: 21058162

[ref63] Thomsen D. K. Jensen A. B. Jensen T. Mehlsen M. Y. Pedersen C. G. Zachariae R. (2013). Rumination, reflection and distress: an 8-month prospective study of colon-cancer patients. Cogn. Ther. Res. 37, 1262–1268. doi: 10.1007/s10608-013-9556-x

[ref64] Threader J. Mccormack L. (2016). Cancer-related trauma, stigma and growth: the “lived” experience of head and neck cancer. Eur. J. Cancer Care (Engl). 25, 157–169. doi: 10.1111/ecc.12320, PMID: 25899673

[ref65] Walshe C. Roberts D. Appleton L. Calman L. Large P. Lloyd-Williams M.. (2017). Coping well with advanced cancer: a serial qualitative interview study with patients and family carers. PLoS One 12, 1–25. doi: 10.1371/journal.pone.0169071, PMID: 28107352 PMC5249149

[ref66] Wang Y. Gan Y. Miao M. Ke Q. Li W. Zhang Z.. (2016). High-level construal benefits, meaning making, and posttraumatic growth in cancer patients. Palliat. Support. Care 14, 510–518. doi: 10.1017/S1478951515001224, PMID: 26481225

[ref67] Wells A. (2000). Emotional Disorders and Metacognition: Innovative Cognitive Therapy. Chichester: John Wiley & Sons.

[ref68] Wells A. Sembi S. (2004). Metacognitive therapy for PTSD: a preliminary investigation of a new brief treatment. J. Behav. Ther. Exp. Psychiatry 35, 307–318. doi: 10.1016/j.jbtep.2004.07.001, PMID: 15530845

[ref69] Wise M. Marchand L. R. Roberts L. J. Chih M. Y. (2018). Suffering in advanced cancer: a randomized control trial of a narrative intervention. J. Palliat. Med. 21, 200–207. doi: 10.1089/jpm.2017.0007, PMID: 29135330 PMC5797325

[ref70] Zhang Y. Xu W. Yuan G. An Y. (2018). The relationship between posttraumatic cognitive change, posttraumatic stress disorder, and posttraumatic growth among Chinese adolescents after the Yancheng tornado: the mediating effect of rumination. Front. Psychol. 9:474. doi: 10.3389/fpsyg.2018.00474, PMID: 29686638 PMC5900041

